# An Analytical Model of Joule Heating in Piezoresistive Microcantilevers

**DOI:** 10.3390/s101109668

**Published:** 2010-11-01

**Authors:** Mohd Zahid Ansari, Chongdu Cho

**Affiliations:** Department of Mechanical Engineering, Inha University, 253 Yonghyun-dong, Nam-Ku, Incheon, 402-751, Korea; E-Mail: ansari.zahid@hotmail.com

**Keywords:** Joule heating, piezoresistivity, microcantilever, bimetallic effect, biosensors

## Abstract

The present study investigates Joule heating in piezoresistive microcantilever sensors. Joule heating and thermal deflections are a major source of noise in such sensors. This work uses analytical and numerical techniques to characterise the Joule heating in 4-layer piezoresistive microcantilevers made of silicon and silicon dioxide substrates but with the same U-shaped silicon piezoresistor. A theoretical model for predicting the temperature generated due to Joule heating is developed. The commercial finite element software ANSYS Multiphysics was used to study the effect of electrical potential on temperature and deflection produced in the cantilevers. The effect of piezoresistor width on Joule heating is also studied. Results show that Joule heating strongly depends on the applied potential and width of piezoresistor and that a silicon substrate cantilever has better thermal characteristics than a silicon dioxide cantilever.

## 1. Introduction

Microcantilever-based sensors have emerged as a powerful, universal and highly sensitive tool to study various physical, chemical, and biological phenomena. They are found to be especially attractive in biochemical and biological sensor applications because of their rapid, label-free and real-time detection abilities [1–7]. The application of microcantilevers in modern sensors was greatly enhanced by the invention of atomic force microscopy (AFM) and the advancements in associated micro-fabrication technologies. The widespread availability of inexpensive micro-fabricated cantilevers has resulted in renewed interest in using surface stress-based cantilever sensors as a means of detecting biomolecule absorption [8].

Microcantilever biosensors exploit surface stress-induced deflections to assay the analyte. The surface stresses, in general, are generated either by the redistribution of the electronic charge at the surface, due to the change in the equilibrium positions of the atoms near the surface, or by the adsorbtion of foreign atoms onto its surface to saturate the dangling bonds [9]. When the target molecules attach onto the functionalized top surface of the cantilever, the surface stress distribution on the surface is changed, resulting in a differential stress across the top and bottom surfaces of the cantilever. The differential stress ultimately generates deflection in the cantilever, whose measurement give information on type and concentration of the analyte. The deflections are usually measure by optical read-out technique. The optical detection technique of deflection measurement in microcantilever sensors has several disadvantages. First, it requires external devices for deflection measurement, *i.e.*, a laser beam and position sensitive detector (PSD), which makes the sensor system bulky and restricts its out-of-lab usage. Second, perfect alignment between laser source, cantilever and PSD is required that necessitates frequent calibration. In addition, the optical properties of the analyte are also critical. If the analyte is translucent or opaque to laser, the electrical signal from the PSD can be diminished significantly. It reduces the resolution of the sensor. These disadvantages can be avoided by integrating the detection elements or devices into the cantilever.

Piezoresistive microcantilevers have shown great potential to be used as sensor in a variety of applications, including strain sensor [10], atomic force microscopy [11], accelerometer [12], microcantilever heater [13], pressure sensor for biomedical application [14] and force sensor [15]. However, it is the biochemical and biosensor applications that are attracting piezoresistive cantilevers most. They have been used as environmental sensor [16], biosensor [17], biochemical sensor [18], in DNA sequencing [19], biomolecular force sensor [20] and immunosensor [21]. Nevertheless, the sensitivity and resolution of piezoresistive detection is generally an order of magnitude less than optical method due to low piezoresistive coefficients and the large noise. Piezoresistor cantilevers are vulnerable to thermal effects such as thermal deflection because of temperature increase by Joule heating. Thus, characterisation of Joule heating in piezoresistive microcantilevers is necessary to improve their accuracy. Recently, Chui *et al*. [22] proposed a highly effective method of reducing thermal sensitivity in piezoresistive sensors by taking advantage of the dependence of the piezoresistive coefficient of silicon on crystallographic orientation.

Piezoresistive microcantilevers were traditionally fabricated from single crystalline silicon substrate with the piezoresistor element created by selectively doping the substrate with a suitable dopant. However, later studies found that for MEMS piezoresistors, polysilicon offers a number of advantages over single-crystalline silicon, including the ability to be deposited on a wide range of substrates [10]. The polycrystalline silicon also exhibits piezoresistivity, but the gauge factor is much smaller than that of single crystalline. Thus, to improve the sensitivity and resolution of piezoresistive microcantilevers, efforts have been made to use soft material cantilever or use single crystalline silicon as piezoresistor to achieve high piezoresistive coefficients [23]. To this use, application of silicon dioxide as substrate and single crystalline silicon as piezoresistor was proposed. However, silicon dioxide microcantilevers fabricated from surface micromachining technology can integrate only polysilicon piezoresistors, which suffer from low piezoresistive coefficients and high noise [21]. In recent days, SOI wafers have been used to fabricate silicon dioxide microcantilevers with etched single crystalline silicon piezoresistors to improve the sensitivity and the resolution [24]. The low Young’s modulus of silicon dioxide combined with the high piezoresistive coefficients of single crystalline silicon piezoresistor presents an ideal solution to improve the sensitivity of piezoresistivity microcantilevers. However, silicon dioxide cantilevers have a major drawback in form of Joule heating produced by the piezoresistor encapsulated inside.

Piezoresistor cantilevers are vulnerable to thermal effects such as the variations in the temperature coefficient of resistance because of change in energy level of the carriers and the thermal deflections because of bimetallic effect. These effects change the characteristics of the piezoresistors significantly. Thaysen *et al.* [25] showed that an increase in temperature to 110 °C increased the fractional resistance of the piezoresistor by about 2%. This is mainly because the increase in temperature increased the TCR of the cantilever. The self-heating characteristics, however, can be exploited to useful effects. For instance, Chui *et al.* [26] and Binnig *et al.* [27] and proposed using the self-heating and self-sensing characteristics of piezoresistive microcantilevers for ultra-high density atomic force microscopy data storage. In a related work, King [28] proposed heated atomic force microscope cantilevers for nanotopography measurements. King *et al.* [29] showed thermal cantilevers have better characteristics than piezoresistive cantilevers in improving the sensitivity of and resolution of AFM topology measurements.

Most of the studies on Joule heating involved experimental and numerical analyses and there are only few analytical models for it. Choudhury *et al.* [30] derived an analytical model for predicting transient self-heating in a piezoresistive cantilever under sinusoidal input voltage. They showed that for 50 Hz input a maximum temperature of about 73 °C is generated within 2 ms. This model neglected the layered structure of the cantilever and assumed a uniform thermal conductivity for the entire cantilever. Yang and Yin [31] included the layered structure in their steady-state analytical model for Joule heating in piezoresistive cantilever, and used thickness ratios for each layer in defining the total thermal conductivity of the cantilever. The model is derived for piezoresistors that show temperature dependence on resistance and therefore requires the temperature coefficient of resistance value for calculations. The present work derives both temperature-independent and -dependent models for self-heating in piezoresistive microcantilevers. To this end, the authors propose the use of volumetric ratio of each layer for defining the total thermal conductivity of the cantilever.

The objective of the present work was to derive a steady-state analytical model for describing the temperature distribution in piezoresistive microcantilever by Joule heating. The model includes the layered structure of the cantilever and uses the effective thermal conductivity calculated from the volumetric contribution of each layer. The results are compared against numerical results obtained using a commercial finite element analysis ANSYS. This work uses two different cantilever materials in the analysis. The cantilevers are made of silicon and silicon dioxide with a p-doped silicon piezoresistor encapsulated within. The effect of applied voltage potential and the piezoresistor width on Joule heating is studied. Thermal deflections produced by Joule heating is compared against the surface stress-induced deflections. And, finally, the effect of cantilever base temperature on total cantilever deflection is studied.

## 2. Theory

Electrical resistivity (*ρ*_e_) is an inherent property of a material to resist the flow of electrical current through it by means of producing electrical resistance. Resistivity is independent of material geometry and depends only on the electronic band structure of the material. The resistivity of a material depends on temperature, given as *ρ*_e_(*T*) = *ρ*_e,0_ (1 + *η* (*T – T_0_*)), where *η* is temperature coefficient of resistivity and *ρ*_e,0_ is the electrical resistivity defined at reference temperature *T_0_*. In general, the resistivity of extrinsic semiconductors and metals increases with temperature but decreases for intrinsic semiconductors.

**Figure 1.**
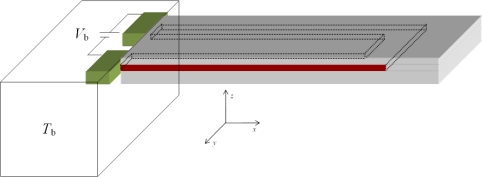
Schematic design of a piezoresistive microcantilever with U-shaped piezoresistor.

Piezoresistivity is the change in bulk electrical resistivity of a material caused by an applied mechanical stress or strain. The resistivity can increase or decrease depending on the material type and the load condition. Many materials exhibit piezoresistivity when strained, but the effect is most pronounced in semiconductors. The piezoresistivity of semiconductors is more than an order of magnitude higher than that of metal. The fractional change in resistance (Δ*R*/*R*) of a piezoresistor due to an externally applied stress is given as [32]:

(1) $$\frac{\Delta R}{R}=\sigma_{l}\pi_{l}+\sigma_{t}\pi_{t}$$

where *σ_l_* and *σ*_t_ and *π*_l_ and *π*_t_ are longitudinal and transverse values of the normal stress and the piezoresistive coefficients. In general, the piezoresistive coefficients are function of dopant type and concentration and the substrate temperature [33]. In fact, it is a decreasing function of concentration and temperature [34]. In biosensor applications, the surface stress-induced deflections produce stress in the cantilever, which change its electrical resistance due to piezoresistivity effect. Thus, the change in resistance provides an indirect way to measure surface stress-induced deflection, and therefore the analyte. The higher the change in Δ*R*/*R* the greater the sensitivity of piezoresistive microcantilever sensors will be. The electrical resistance and its variation, nevertheless, are also responsible for Joule heating which is a major source of noise in piezoresistive microcantilever sensors. Figure 1 shows the schematic design of a typical piezoresistive microcantilever. The U-shaped piezoresistor element (in red) is encapsulated in the substrate and normally bias voltage between 5 and 10 V is applied across it. A thin film of gold on top is generally applied to study cases involving chemical, biochemical or biological samples.

Joule heating is an energy dissipation phenomena commonly observed in electrical current carrying conductors. It converts irreversibly the electrical energy to thermal energy. It is also known as Ohmic heating or electrical-resistance heating. Most of thermal energy is generated due to loss of kinetic energy of current carrying electrons by collisions among themselves and with the lattice atoms. The volumetric rate of Joule heating (W/m^3^) can be given as [35]:

(2) $${\dot{s}}_{J}=\frac{V_{b}^{2}}{\rho_{e}L^{2}}$$

where *V*_b_ is applied electrical potential and *L* is length of conductor. The heat conduction equation predicting the Joule heating effect in piezoresistive microcantilevers is derived next.

The differential-volume thermal energy conservation equation relating heat conduction, convection and radiation to heat storage/dissipation and energy conversion can be expressed as [35]

(3) $$\nabla \cdot q\equiv \nabla \cdot (-k\nabla T+\rho c_{p}\mathrm{Tu}+q_{r})=-\frac{\partial \rho c_{p}T}{\partial t}+\sum_{i}{\dot{s}}_{i}$$

where *q* is heat flux, *k* is thermal conductivity, *ρ* is mass density, *C*_p_ is heat constant, *u* is fluid flow, *q*_r_ is radiation heat flux and *t* is time. The last term includes the energy conversions due to change in chemical- and physical-bond energy, electromagnetic and mechanical characteristics.

Piezoresistive microcantilevers are operated in either liquid or gaseous media. However, due to very small size and high volumetric rate of heat energy generation due to Joules heating, the heat conduction within the microcantilever structure will dominate the heat transfer to liquid or gaseous media via convection or radiation. Thus, the heat conduction mode is the most relevant. Neglecting the convection and radiation modes of heat transfer and considering Joule heating as the only energy conversion, Equation (3) can be modified as:

(4) $$-\nabla \cdot k\nabla T=-\frac{\rho c_{p}\partial T}{\partial t}+{\dot{s}}_{J}$$

This is the most general three-dimensional form of heat conduction equation including thermal energy generation. Assuming thermal conductivity is weak function of temperature, Equation (4) in Cartesian coordinates can be given as:

(5) $$\frac{\partial^{2}T}{\partial x^{2}}+\frac{\partial^{2}T}{\partial y^{2}}+\frac{\partial^{2}T}{\partial z^{2}}=\frac{1}{\alpha }\frac{\partial T}{\partial t}-\frac{{\dot{s}}_{J}}{k}$$

where *α* (= *k/ρC*_p_) is the thermal diffusivity. This equation is also known as heat diffusion equation. The thermal diffusivity is the controlling transport property for transient conduction. The higher the value of thermal diffusivity the faster the system will reach its new temperature equilibrium.

Since piezoresistive microcantilevers have slender shape and the piezoresistor is symmetric along the path of electrical current, the temperature rise due to Joule heating will remain uniform along width and thickness direction (Figure 1). The temperature profile will, however, change along the cantilever length. In other words, the heat conduction can be assumed one-dimensional. Since the value of thermal diffusivity is very high for the microcantilevers, the system will reach steady state very rapidly. Thus, the heat conduction in microcantilevers can be assumed one-dimensional and steady state case. And Equation (5) can now be combined with Equation (2) to give:

(6) $$\frac{\partial^{2}T}{\partial x^{2}}=-\frac{1}{k}\frac{V_{b}^{2}}{\rho_{e,0}L^{2}}$$

**Figure 2.**
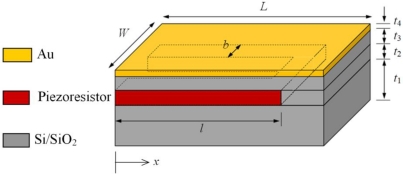
Typical geometry of a 4-layer piezoresistive microcantilever.

Figure 2 shows the geometric properties of a typical 4-layer piezoresistive microcantilever with U-shaped piezoresistor. In piezoresistive microcantilevers, the piezoresistor is encapsulated in the substrate to achieve electrical insulation; and the electrical current is therefore confined to within the piezoresistor element. The heat, nevertheless, will spread rapidly to the entire cantilever structure by conduction. Since piezoresistor is the only current carrying medium in the microcantilever, the Joule heating will occur only in the piezoresistor. Therefore, the amount of heat generated will depend only on the cross-sectional area (*A*_pzr_ = *b* × *t*_2_) and centreline length (*L*_pzr_ = 2*l + W – 2b*) and the electrical resistivity of the piezoresistor. The negative sign in Equation (6) can now be omitted because traditionally this sign was used to indicate the heat loss in a system, but in our case the heat is generated. Thus, Equation (6) in term of piezoresistor heating per unit cantilever volume can be given as:

(7) $$\frac{\partial^{2}T}{\partial x^{2}}=\frac{1}{k_{\mathrm{eff}}}\frac{V_{b}^{2}A_{\mathrm{pzr}}}{\rho_{e,0}L_{\mathrm{pzr}}V}$$

where *V* is volume of the cantilever and *k*_eff_ is the effective thermal conductivity of the 4-layer microcantilever structure. Since the layer containing the piezoresistor is discontinuous, use of thermal resistance in series or parallel combination to know heat transfer is unhelpful. The heat flow is anisotropic and three-dimensional. Therefore, effective conductivity is the best way to define thermal behaviour. The effective thermal conductivity can be calculated by applying the rule of mixtures, and is given as $k_{\mathrm{eff}}=\sum_{i=1}^{4}n_{i}k_{i}$, where *n* is volume fraction of each material and *k* its thermal conductivity.

Applying the boundary conditions *T*|_x=0_ = *T_b_* and the adiabatic condition ${\frac{\mathrm{dT}}{\mathrm{dx}}|}_{x=0}=0$ at the base, Equation (7) gives:

(8) $$T(x)=T_{b}+\frac{1}{2k_{\mathrm{eff}}}\frac{V_{b}^{2}A_{\mathrm{pzr}}x^{2}}{\rho_{e,0}L_{\mathrm{pzr}}V}$$

and the maximum temperature as:

(9) $$T_{\text{max}}(x=L)=T_{b}+\frac{1}{2k_{\mathrm{eff}}}\frac{V_{b}^{2}A_{\mathrm{pzr}}L^{2}}{\rho_{e,0}L_{\mathrm{pzr}}V}$$

The above equation is temperature-independent because it does not require the temperature coefficient of resistance to calculate the temperature variation in the cantilever. The temperature-dependent form is derived next.

The temperature-dependent form of Equation (7) in form of current density (*J*) can be written as:

(10) $$\frac{\partial^{2}T}{\partial x^{2}}=\frac{J^{2}V_{\mathrm{pzr}}}{k_{\mathrm{eff}}V}\rho_{e}(T)$$

where *V*_pzr_ is volume of the piezoresistor element and *ρ*_e_(*T*) = *ρ*_e,0_ (1 + *η* (*T – T*_0_)) its temperature dependent resistivity. Using identical boundary conditions as above, the resistivity relation can now be combined with Equation (10) and solved to give the temperature-dependent form as:

(11) $$T(x)=\frac{T_{b}}{2}\left[e^{\sqrt{C_{1}x}}+e^{-\sqrt{C_{1}x}}\right]+\frac{C_{2}x^{2}}{2}$$

where:

$$C_{1}=\frac{\rho_{e,0}\eta J^{2}V_{\mathrm{pzr}}}{k_{\mathrm{eff}}V}\text{ and }C_{2}=\frac{\rho_{e,0}(1-\eta T_{0})J^{2}V_{\mathrm{pzr}}}{k_{\mathrm{eff}}V}$$

or, in terms of bias voltage *V*_b_:

$$C_{1}=\frac{\eta V_{b}^{2}A_{\mathrm{pzr}}}{\rho_{e,0}k_{\mathrm{eff}}L_{\mathrm{pzr}}V}\text{ and }C_{2}=\frac{(1-\eta T_{0})V_{b}^{2}A_{\mathrm{pzr}}}{\rho_{e,0}k_{\mathrm{eff}}L_{\mathrm{pzr}}V}$$

and the maximum temperature in the cantilever is:

(12) $$T_{\text{max}}(x=L)=\frac{T_{b}}{2}\left[e^{\sqrt{C_{1}L}}+e^{-\sqrt{C_{1}L}}\right]+\frac{C_{2}L^{2}}{2}$$

It can be observed from Equation (12) that if the resistivity coefficient *η* is very low or zero, the equation will reduce to its temperature-independent form shown by Equation (9). Since the typical value of *η* for p-doped silicon is 1 × 10^−4^ °C, a temperature increase of 100 °C will result in about 1% increase in resistivity. Therefore, we suggest Equation (9) should be used for temperatures below 100 °C. This equation is used in the present study because the maximum temperature generated is about 64 °C.

In deriving above equations certain assumptions were made. We assumed that adiabatic condition exists at the base and the temperature of the base is unchanged. This point is explained later in this paper. We also assumed there is no thermal contact resistance between the layers and there is perfect bonding between the layers. The material properties remain unchanged. In the next section, the temperature profile predicted by Equation (9) will be compared against the results obtained from numerical analysis.

## 3. Numerical Analysis

A commercial finite element analysis (FEA) software ANSYS Multiphysics v.11 was used to numerically analyse the temperature distribution and thermal deflection in the microcantilevers due to Joule heating. The effect of bias voltage and piezoresistor width was also investigated. The voltage was varied from 5 to 10 V and the width was changed to 15 μm, 30 μm, and 45 μm. The FE model was meshed by 3-D coupled field 8-node scalar SOLID5 elements and was solved under steady-state condition. These elements have capability to perform coupled problems involving mechanical, thermal, electrical and piezoresistive effects. About 100,000 elements were used in each analysis. Table 1 lists the geometric properties of the 4-layer piezoresistive microcantilever model shown in Figure 2.

Piezoresistive microcantilevers of silicon and silicon dioxide substrate were analysed. In case of silicon cantilever, the substrate is made of silicon and the piezoresistor is made of doped silicon. Similarly, in case of silicon dioxide cantilever, the substrate is made of silicon dioxide and the piezoresistor is made of doped silicon. The geometric properties of both silicon and silicon dioxide microcantilevers are same. Typical material properties of constituent layers are listed in Table 2.

In the numerical analysis, a first analysis was performed to validate the theoretical model derived in previous section, *i.e.*, Equation (9). To this use, the effect of increase in bias voltage on maximum temperature generated in the silicon and silicon dioxide cantilevers was studied. In numerical analysis, the finite element model nodes representing the cantilever base, *i.e.*, the entire left face of the cantilever shown in Figure 2, were kept at 25 °C and no additional temperature boundary conditions were applied. These conditions are the same used in deriving the analytical model. After validation, the effect of the change in piezoresistor width and the change in bias voltage on maximum temperature and maximum thermal deflection was investigated. In total, six voltages and three width values were used. Other geometric properties were kept constant. The cantilever models were then investigated for effect of base temperature induced deflections with no voltage applied. In this case, the temperature of the cantilever was equal to the base temperature of 25 °C. Finally, surface stress-induced deflections were determined. Herein, no temperature and voltage boundary conditions were applied. The analysis assumed that temperature has negligible effect on the properties of the cantilever.

## 4. Results and Discussion

**Figure 3.**
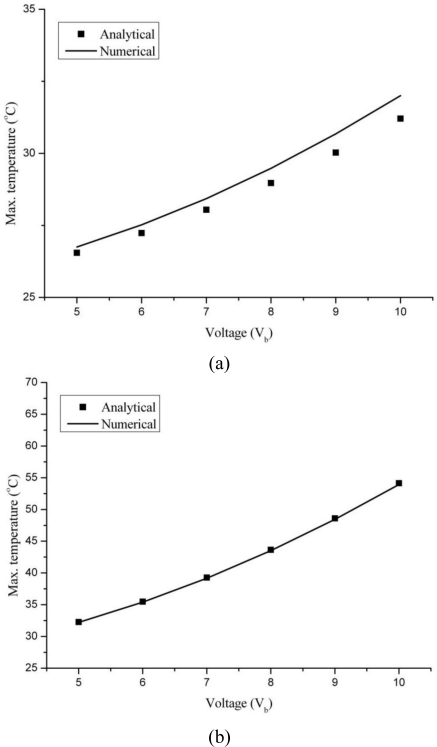
Comparison between analytical and simulation results for silicon **(a)** and silicon dioxide **(b)** piezoresistive microcantilevers for *b* = 30 μm and *T*_b_ = 25 °C.

Figure 3 presents the comparison between analytical and numerical results for maximum temperature at different applied voltages for silicon and silicon dioxide cantilevers. The analytical results were determined using Equation (9). The comparison results are showing good accord in predicting the maximum temperature value; and therefore indicating the validity of Equation (9). Moreover, as suggested by Equation (9), the temperature and applied voltage is showing a parabolic dependence. The good correlation between analytical and numerical results also verifies the assumption that the thermal conductivity of the 4-layer microcantilever structure can be predicted using the rule of mixtures. The effective thermal conductivity of the silicon and silicon dioxide cantilevers are calculated as 161.13 × 10^6^ and 34.31 × 10^6^ pW/μm °C, respectively.

Based on the results shown in Figure 3, we can conclude that the temperature variations due to Joule heating in piezoresistive cantilevers can be predicted by Equation (9). The equation indicates that temperature variation depends only on geometric and thermo-electric properties of the cantilever materials and the cantilever base temperature. It does not, however, depend on their mechanical properties. The temperature at cantilever base is important. As can be seen in Figure 3, the base temperature contributes nearly 78% and 46% to the maximum temperatures generated in silicon and silicon dioxide cantilevers, respectively. Thus, maintaining the base temperature at a lower value can help in greatly reducing the temperature rise in piezoresistive microcantilevers. Further, an observation can be made regarding electrical resistivity and Joule heating that a piezoresistor of low electrical resistivity will generate more Joule heating than one with high resistivity. In addition, long and thin piezoresistor obviously can also help in this regard.

The maximum deviation in the analytical and numerical results for silicon and silicon dioxide are about 2.5% and 0.4%, respectively. The deviation can be attributed to the assumptions made in deriving the analytical model. The analytical model assumed one-dimensional heat flow, but in numerical analysis it is a three-dimensional problem. In addition, the shape of piezoresistor is not considered in the analytical model. The shape is crucial in distribution of temperature in the cantilever. The numerical analysis used a U-shaped piezoresistor and there can be non-uniform temperature distribution in the cantilever. Another reason for deviation can be the use of effective thermal conductivity, which assumed the entire cantilever has a constant thermal conductivity. But in numerical analysis, the cantilever is a layered structure of materials having different thermal conductivities. Nevertheless, the low deviation between the analytical and numerical result provides a simple and useful analytical relation for estimating the Joule heating in a complicated microcantilever structure.

In deriving Equation (9), it was assumed that base temperature remains unchanged and adiabatic condition can be used. This assumption is reasonable in view of the fact that the size and volume of the base is extremely large than that of the microcantilever. The large size of the base provides large thermal mass. Thermal mass is an extrinsic property of the body and is calculated as the product of mass of the body and its heat capacity. Thermal mass measures the ability of the body to resist the temperature variations. Thus, a body with large mass or large heat capacity will also have a large thermal mass. And, therefore large amount of heat energy will be required to change its temperature. In general, the volume of base is extremely large, only large amounts of Joule heating can alter its temperature considerably. During normal operations of piezoresistive microcantilevers, the amount of heat generated due to Joule heating is insufficient to cause any significant change in the base temperature. Therefore, we can conclude that the base temperature of the cantilever will remain relatively same and the adiabatic condition can be used.

**Figure 4.**
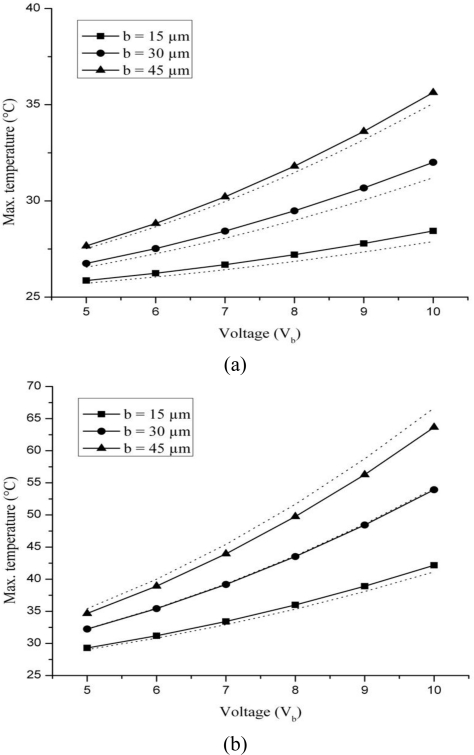
Effect of bias voltage on maximum temperatures generated in silicon **(a)** and silicon dioxide **(b)** microcantilever for different piezoresistor width. Dotted lines show analytical results.

Figure 4 shows the comparison between maximum temperature results obtained from numerical analysis for silicon and silicon dioxide cantilevers having piezoresistor of widths 15, 30 and 45 μm. The results obtained from the analytical relation are also plotted for a comparison. The analytical results are shown by dotted lines. The maximum deviation between analytical and numerical results for silicon and silicon dioxide cantilever are 1.9%, 2.5% and 1.5% and 2.4%, 0.4% and 4.5%, respectively, for piezoresistor widths 15, 30 and 45 μm. It is obvious from the figure that the increase in applied voltage and width both increased the temperature. These observations agree with the predictions by Equation (9) that temperature is directly proportional to voltage and width. The width term included in defining the current carrying length, *i.e.*, *L*_pzr_, has no appreciable effect on temperature results.

It is further observed in Figure 4 that the maximum temperature generated in a silicon dioxide cantilever is more than 1.5 times that in a silicon cantilever. Since the amount of Joule heating depends only on the conditions of piezoresistor, which is same in both the cases, the amount of heat generated in silicon and silicon dioxide cantilevers should be same. The difference between the temperatures in silicon and silicon dioxide is because of different thermal conductivities of these materials. The thermal conductivity of silicon is more than 100 times that of silicon dioxide. The higher the thermal conductivity, the greater the heat diffusion will be. In other words, if thermal conductivity of a material is large, there will be more temperature uniformity in the material. Since the thermal conductivity of silicon dioxide is much lower than silicon, there will be more non-uniformity in it. Therefore, we may conclude that the maximum temperature observed in silicon dioxide is high because of its low thermal conductivity. This behaviour is also suggested by Equation (9) that indicates an inverse relation between temperature and thermal conductivity. Thus, the temperature rise due to Joule heating will be higher in cantilevers made of low thermal conductivity materials.

**Figure 5.**
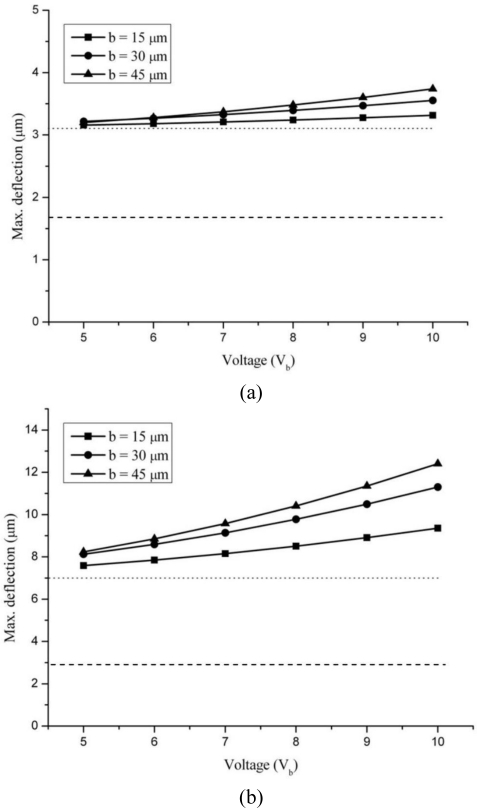
Effect of bias voltage on maximum deflections in silicon **(a)** and silicon dioxide **(b)** cantilevers of different piezoresistor widths.

Figure 5 presents the numerical results for deflection behaviour of silicon and silicon dioxide cantilevers due to Joules heating. The dotted line shows the deflection for *V*_b_ = 0 and *T*_b_ = 25 °C and the dashed line for surface stress *σ_s_* = 1 N/m and *T*_b_ = 0. The Joule heating-induced deflections are produced by the bimetallic effect, which, in turn, arises due to different coefficients of thermal expansion (CTEs) of the constituent layers of the silicon and silicon dioxide cantilevers. The mismatch in their CTEs results in thermal strain, causing deflections in the structure. In our case, the deflections increase with increase in bias voltage and show a nonlinear dependence similar to that for temperature. In fact, the higher the temperature, the higher the deflections will be. Thus, as the voltage is increased the temperature is also increased because of Joule heating, resulting in increasing the thermal deflection.

The thermal deflections of Figure 5 are generated largely by the mismatch between the CTE of silicon and silicon dioxide substrates and the gold film. In addition, the Young’s modulus of the substrates also plays a critical role. Substrates made of low Young’s modulus materials with deflect easily than one with high modulus. Thus, the higher deflections observed in silicon dioxide cantilever can be attributed to the combined effect of higher temperature due to Joule heating and its low Young’s modulus. In all the cases analysed, the cantilevers bend downwards. The deflections were found to increase with the increase in piezoresistor widths, which is understandable because wide piezoresistors generate more heat.

Interestingly, thermal deflections are observed in both silicon and silicon dioxide cantilevers for cases when no voltage is applied, *i.e.*, *V*_b_ = 0. This deflection is shown by dotted lines in Figure 5. The origin of this deflection can be attributed to bimetallic effect generated due to initial temperature of the cantilever structure. In this particular analysis, the temperature of the entire structure including base and cantilever was 25 °C. The temperature combines with the CTE mismatch between constituent layers of the microcantilever to induce bimetallic effect and leads to thermal deflections. In fact, the initial temperature alone is responsible for more than 50% of total temperature in both cases. We also found that for *V*_b_ = 0, the increase in width has no significant effect on deflection, and the three deflection curves for width overlie. Thus, the base temperature should be kept low to reduce errors.

The dashed lines in Figure 5 represent the numerical results for deflections induced only by the surface stress in the cantilevers. A two-dimensional surface stress of 1 N/m was applied to their top surfaces. In numerical analysis, the surface stress is modelled as tensile force applied to the top surface of the cantilever [36]. It is observed that for same surface stress, the deflections in silicon cantilevers are less than half the silicon dioxide cantilevers. This is because of the higher Young’s modulus of the silicon, which is more than two times that of silicon oxide. Further, it is obvious from the figure that thermal deflections exceed the surface stress-deflections in all the cases. We also found that change in piezoresistor width has no appreciable effect on surface stress-induced deflection, and the three deflection curves for width overlie. Since the thermal- and surface stress-induced deflections occur independently and therefore can be superimposed to obtain the total deflection [37]. Thus, by knowing the base temperature and the temperature due to Joule heating, the surface stress-induced deflection can easily be isolated and determined from total deflection.

The rise in temperature in piezoresistive microcantilevers by Joule heating produces different effects in the cantilever. First, it causes bimetallic effect in the cantilever that produces thermal deflection-induced resistance change in the cantilever; second, it causes variation in the electrical resistance of the piezoresistor given by coefficient *η*; and third, it also causes variations in the piezoresistive coefficient of the piezoresistor given by coefficient *φ*. The total relative change in the resistance of the piezoresistor, which is also termed as its total sensitivity, is a combination of all the above effects, and by knowing the increase in temperature the relative contribution of each can be determined. The mechanical properties of the cantilever are, however, less affected by temperature.

Since the sensitivity of a piezoresistive microcantilever sensor is defined by the fractional change in resistant occurred, the deflection and temperature sensitivities of the piezoresistive sensor can be given as [21]:

(13) $$\frac{\Delta R_{\mathrm{def}}}{R}=\frac{3\pi_{l}E_{l}d}{2L^{2}}\Delta z$$

(14) $$\frac{\Delta R_{\mathrm{temp}}}{R}=\eta \Delta T$$

where *E*_l_ is longitudinal elastic modulus of cantilever, *d* is the distance between piezoresistor axis and the neutral axis of the cantilever and Δ*z* is deflection. Equations (13) can be used for calculating the sensitivity of the cantilever to both thermal- and surface stress-induced deflections, and Equation (14) is used for change in cantilever resistant due to temperature variation. The summation of both equations gives the total thermal sensitivity. In this study, *E*_l_ = 160 × 10^3^ MPa and *d* = 0.172 μm and 0.156 for both silicon and silicon dioxide cantilevers. The typical values of *π_l_*, *η* and *φ* for p-doped silicon are 72 × 10^−11^ Pa^−1^, 1 × 10^−4^ °C and −27 × 10^−4^/°C, respectively [25,26]. The negative coefficient of *φ* indicates piezoresistivity decreases with increase in temperature. Therefore, Equation (13) is also temperature dependent with *π*_l_ (*T*) = *π*_l,0_ (1 – 27 × 10^−4^ Δ*T*).

**Figure 6.**
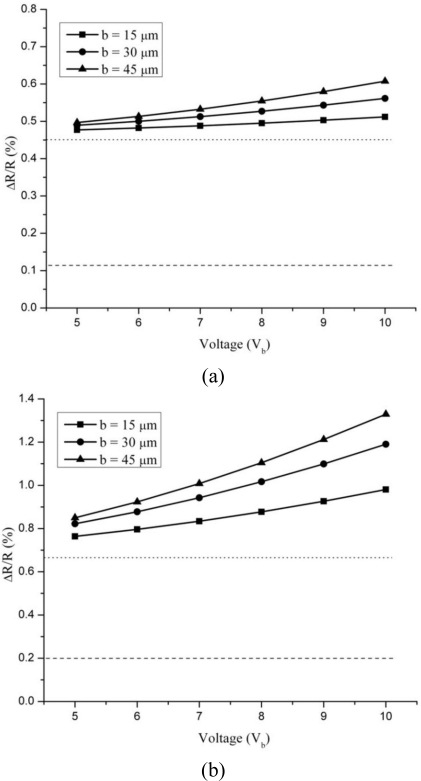
Total thermal sensitivity of silicon **(a)** and silicon dioxide **(b)** cantilevers.

Figure 6 presents the effect of bias voltage on total thermal sensitivity of the silicon and silicon dioxide cantilevers. For a comparison, the sensitivity value for surface stress is also plotted. The dotted and dashed lines represent the sensitivity values for base temperature and surface stress, respectively. The deflections caused by Joule heating, by cantilever base temperature and by surface stress were determined separately (see Figure 5), and therefore their sensitivities were also determined separately and then combined for obtaining the total sensitivity results. The total sensitivity for the first two cases was calculated by addition of Equations (13) and (14); this also included the variation in piezoresistive constant. However, in the last case the surface stress sensitivity was determined from Equation (13) alone because thermal effects were not considered. All the temperature and deflection values used are adopted from the numerical results shown in Figures 4 and 5.

It is obvious in Figure 6 that the total thermal sensitivity of both silicon and silicon dioxide cantilevers to Joule heating effects increases with an increase in voltage, and the increase is more pronounced in the latter. This observation is very closely related to the results for temperature increase shown in Figure 5, wherein silicon dioxide cantilevers produced higher values for temperatures and deflections. The high voltages produce high temperatures in the cantilevers and result in high deflections. In addition, high temperatures decrease the piezoresistive coefficient. Thus, the result of increase in temperature on piezoresistive microcantilevers can be summarised as increase in resistance, increase in thermal deflection and decrease in piezoresistive constant.

The surface stress sensitivity results for silicon and silicon dioxide cantilevers are 11.04 × 10^−4^ and 20.56 × 10^−4^, respectively. These values which are the signal are lower than those produced due to thermal effects which are the noise. This suggests a low signal-to-noise ratio (S/N) for the cantilever and that the cantilever is more sensitive to Joule heating effects than to surface stress effects. Nevertheless, the sensitivity values for deflection sensitivity are of same order of magnitude of Joule heating effects. Since low voltages produce less thermal effects, temperature rise should be controlled for optimum sensor performance. Thus, the sensitivity of the cantilever to Joule heating effects can be reduced by applying low voltages. Since low voltages also reduce the current flow which will decrease the S/N, a more practical approach is to use symmetrical Wheatstone bridge and differential read-out to eliminate Joule heating-induced thermal effects in the piezoresistive microcantilever sensor.

**Figure 7.**
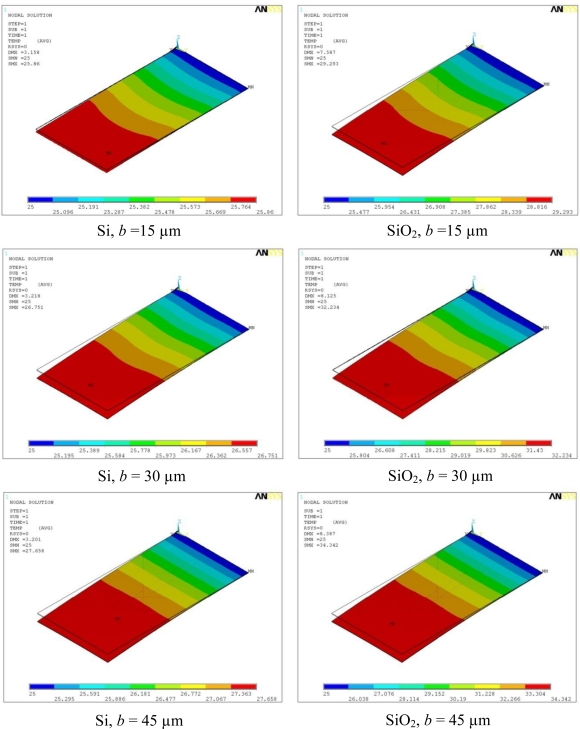
Temperature profile in silicon and silicon dioxide piezoresistive microcantilevers at *V*_b_ = 5 V.

The temperature distribution in silicon and silicon dioxide piezoresistive microcantilevers for bias voltage of 5 V is presented in Figure 7. The minimum temperature in all the models is the base temperature of 25 °C, and occurs near the base region. The maximum temperature occurs near the tip region of the cantilever and its magnitude depends on the cantilever property. The figure clearly demonstrates the fact that temperature distribution in the microcantilevers is one-dimensional, because it depends only on the distance along the cantilever length. In addition, the distribution is symmetric along the length. The maximum (SMX) and minimum (SMN) temperatures and the corresponding thermal deflection (DMX) values are mentioned in the top-left corner of the micrographs. The un-deformed shape is indicated by dotted edge. The deformed shapes are for illustration only and are not to scale. It can also be observed in the figure that for each cantilever type, the increase in piezoresistor width increases the maximum temperature zone of the cantilever. In other words, the cantilever area subject of maximum temperature increases with the increase in piezoresistor width. This is because increase in piezoresistor width also increases the Joule heating and the area generating heat is therefore also increased.

## 5. Conclusions

Characterisation of Joule heating in piezoresistive microcantilevers is necessary to improve their sensitivity. To this end, the present work investigated the effect of applied voltage and the piezoresistor width on temperature distribution and thermal deflections in the cantilevers. This work developed a theoretical model to predict the temperature distribution in piezoresistive microcantilevers due to Joule heating. Results showed that low bias voltage should be applied for reducing Joule heating. Further, since temperatures in cantilevers depend directly on the width of piezoresistor element, narrow elements should be used for reducing Joule heating. Numerical results showed that because of their high thermal conductivity silicon cantilevers generate less heat than silicon dioxide ones. Therefore, silicon cantilevers or cantilevers made of high thermal conductivity material should be used to reduce Joule heating. The temperature rise in cantilevers induced bimetallic effect and produced thermal deflection, which are a major source of noise. We found the temperature of the cantilever base plays a critical role in temperature rise in the cantilever, and should be maintained at low temperatures for minimising the thermal noise. Future works will focus on thermal stress characteristics of piezoresistive microcantilevers due to Joule heating.

## Figures and Tables

**Table 1.** Geometric properties of piezoresistive microcantilevers.

Length of cantilever, *L*	200 μm
Width of cantilever, *W*	100 μm
Length of piezoresistor, *l*	180 μm
Width of piezoresistor, *b*	15, 30, 45 μm
Thickness of substrate, *t*_1_	0.5 μm
Thickness of piezoresistor, *t*_2_	0.1 μm
Thickness of insulation, *t*_3_	0.1 μm
Thickness of gold film, *t*_4_	0.05 μm

**Table 2.** Material properties of piezoresistive microcantilevers in μMKS units.

**Parameter**	**Si**	**Au**	**SiO_2_**
Elastic modulus, *E* (MPa)	160 × 10^3^	80 × 10^3^	70 × 10^3^
Poisson’s ratio, *ν*	0.23	0.42	0.20
Mass density, *ρ* (kg/μm^3^)	2.32 × 10^−15^	19.3 × 10^−15^	2.22 × 10^−15^
Electrical resistivity, *ρ*_e_ (Tohm-μm)	1 × 10^−9^	----	----
Thermal conductivity, *k* (pW/μm °C)	150 × 10^6^	317 × 10^6^	1.38 × 10^6^
Thermal expansion coefficient, *λ* (1/°C)	2.8 × 10^−6^	14.2 × 10^−6^	0.5 × 10^−6^
Specific heat, *c*_p_ (PJ/kg °C)	712 × 10^12^	129 × 10^12^	745 × 10^12^
